# Strategies and rules for tuning TCR-derived therapy

**DOI:** 10.1017/erm.2023.27

**Published:** 2023-12-14

**Authors:** Guoheng Mo, Xinyu Lu, Sha Wu, Wei Zhu

**Affiliations:** 1 State Key Laboratory of Organ Failure Research, Guangdong Provincial Key Laboratory of Viral Hepatitis Research, Department of Infectious Diseases, Nanfang Hospital, Southern Medical University, Guangzhou, China; 2 Department of Immunology/Guangdong Provincial Key Laboratory of Proteomics, School of Basic Medical Sciences, Southern Medical University, Guangzhou 510515, People's Republic of China

**Keywords:** affinity maturation, bond engineering, cell engager, immunotherapy, modification strategy, TCR-T, tumour microenvironment

## Abstract

Manipulation of T cells has revolutionized cancer immunotherapy. Notably, the use of T cells carrying engineered T cell receptors (TCR-T) offers a favourable therapeutic pathway, particularly in the treatment of solid tumours. However, major challenges such as limited clinical response efficacy, off-target effects and tumour immunosuppressive microenvironment have hindered the clinical translation of this approach. In this review, we mainly want to guide TCR-T investigators on several major issues they face in the treatment of solid tumours after obtaining specific TCR sequences: (1) whether we have to undergo affinity maturation or not, and what parameter we should use as a criterion for being more effective. (2) What modifications can be added to counteract the tumour inhibitory microenvironment to make our specific T cells to be more effective and what is the safety profile of such modifications? (3) What are the new forms and possibilities for TCR-T cell therapy in the future?

## Background

TCR-T therapy (T-cell receptor T-cell therapy) is a cutting-edge immunotherapy approach designed to treat various types of cancers. It involves genetically modifying a patient's own T-cells to express special receptors called T-cell receptors (TCRs) that can recognize and target cancer cells. These modified T-cells are then expanded in the laboratory and infused back into the patient's body. The TCRs enable the T-cells to identify specific antigens present on the surface of cancer cells, initiating a targeted immune response to eliminate the cancer. TCR-T therapy holds promise in the field of cancer treatment by harnessing the power of the immune system to target and destroy cancer cells with precision, potentially offering a personalized and effective treatment option for patients who may not respond well to conventional therapies. More information concerning cloning methods and treatment process of TCR-T can be found in these two reviews (Refs 1, 2). TCR-T alone cannot meet the therapeutic requirements for solid tumours. Drawing from prior experience, TCR sequences frequently undergo targeted affinity maturation and other enhancements to T-cell efficacy before TCR-T therapies progress to clinical stages. For these aspects, the article will be developed in detail.

## The necessity of affinity modification

The recognition of target antigens and the consequential cytotoxic functionality of T cells hinges upon the critical factor of the TCR affinity (Ref. 3). The extent of cytokine release and cytotoxicity is typically influenced by TCR affinity as well (Refs 4, 5). In vitro, design strategies have been explored to obtain mature TCRs with optimized affinity, which have demonstrated superior anti-tumour activity in co-culture experiments. Additionally, animal models have provided evidence that T cells possessing high-affinity TCRs are more efficacious in facilitating tumour regression (Refs 6, 7).

The optimization of TCRs with heightened affinity represents a promising avenue for developing efficacious immunotherapies to combat cancer (Ref. 8). This alteration is based on the concept of the therapeutic window applicable to tumour-specific or virus-specific antigens. This concept provides a chance to enhance the binding affinity of TCRs towards particular antigens. Nevertheless, the effectiveness and safety of T-cell immunotherapy rely on carefully choosing TCRs that possess suitable affinity ranges (Ref. 9). The affinity of TCRs must be meticulously scrutinized to ensure successful tumour regression without engendering unexpected autoimmune responses. It has been reported that TCRs with low affinity fail to engender sufficient antitumour effects in vitro and in vivo, whereas T cells with high affinity are commonly linked to autoimmune responses (Ref. 8). As such, it is imperative to ascertain a judicious range for affinity modification, contingent on the specific antigens, the morphological manifestation of the tumour (haematological or solid) and the type of tumour itself.

At present, most TCR-T cell products currently known have undergone affinity optimization and modification before entering clinical validation. Firstly, natural TCRs exhibit relatively low affinity. Tumour-specific TCRs selected by the thymus at the micromolar range have lower affinity for peptide major histocompatibility (pMHC) complex (Refs 5, 10, 11) particularly for self-tumour-associated antigens (Ref. 12). Secondly, the immune evasion mechanism of tumours results in the downregulation of antigen density. T-cell activation is contingent on the binding kinetics of TCR–pMHC, which is influenced by the density of tumour cells or antigen-presenting cells (APCs) on the membrane (Ref. 10). Nonetheless, cancer cells generally present epitopes with low density to evade immune surveillance, which culminates in defects in antigen processing and presentation mechanisms (Ref. 13). Thirdly, the suppressive effect of the tumour microenvironment (TME) and the drug resistance mechanism of solid tumours has led to the unsatisfactory clinical efficacy of adoptive cell therapy. In particular, after the treatment of CAR-T(Chimeric Antigen Receptor T) and TCR-T shifted from haematological to solid tumours, most solid tumours present a fibrotic stroma, which includes an abundant extracellular matrix, regarded as the main mechanism of resistance (Refs 14, 15). Taken together, these factors underscore the need for higher TCR affinity than natural TCRs in current clinical treatments (Refs 16, 17) In conclusion, the maturation of TCR affinity is crucial for achieving optimal therapeutic effects. Improving TCR affinity can overcome challenges such as the reduction in antigen density, the suppressive impact of the TME and the drug resistance mechanisms present in solid tumours.

### Parameters and standards for affinity engineering

#### Affinity in 2D and 3D

The affinity between the receptor (TCR) and ligand (pMHC) is a critical factor in predicting T-cell responses to antigens. This affinity, or binding constant (Ka), is determined by the equilibrium between the receptor and ligand and can be calculated using the binding rate (Kon) and dissociation rate (Koff), regardless of ligand concentration or receptor calculations (Ref. 18). The dissociation constant and half-life can also be calculated (Ref. 19). The assessment of affinity values is typically accomplished through surface plasmon resonance (SPR) in solution using purified reactants. In this technique, the free ligand flows over the receptor immobilized on the surface to measure absolute affinity in a three-dimensional space, providing a definition of protein interactions in their purest form, free from external forces. It has been observed that TCR affinity does not always correlate with T-cell function, possibly due to fundamental differences in biophysical characteristics between antibody–antigen interactions and TCR antigen recognition (Refs 5, 10). While antibodies are designed to bind to entire antigen proteins in a 3D fluid phase, leading to high affinity and long bond lifetimes even at zero force, TCRs function as transmembrane proteins with low 3D affinity and rapid off-rates in force-free conditions for pMHC (Refs 20, 21).

Techniques such as flow chamber analysis, thermal fluctuation analysis, single-molecule Förster resonance energy transfer, Zhu–Golan plot, contact area fluorescence recovery after photobleaching and adhesion frequency assays provide 2D receptor–ligand binding methods that better replicate membrane surface protein interactions (Ref. 18). This type of affinity is referred to as relative affinity, as it depends on the specific environment in which it is measured. In contrast, 3D methods generate absolute affinity measurements but fail to account for the influence of other cellular components (Ref. 22). The use of this method to measure TCR–pMHC affinity is an extremely sensitive approach that follows first-order kinetics and is dependent on intrinsic T-cell factors. Additionally, these 3D methods do not consider the impact of reduced molecular motion space or the presence of a co-receptor (Ref. 23). In contrast, 2D receptor–ligand binding techniques have shown a better correlation with in vitro T-cell functional responses (Refs 24, 25). In a clinical trial involving T-cell transduction with autologous melanoma reactive TCR, it was found that patients who had ideal treatment responses had a higher 2D affinity for the engineered T cells compared to non-responders, despite having similar 3D affinity measurements (Ref. 26). Therefore, we recommend prioritizing 2D affinity measurements when selecting experiments to measure affinity values, as the relative affinity measured by 2D methods has a stronger correlation with functional responses compared to 3D methods.

#### Avidity and functional avidity

The advent of recombinant pMHC tetramer reagents has revolutionized the identification and screening of antigen-specific T cells in vitro. This approach indirectly evaluates the biophysical interaction between TCR and pMHC and takes into account the influence of other co-receptors, such as CD8 receptors, during the interaction. The binding of one pMHC monomer arm enhances the Kon of the subsequent monomer arm and reduces the koff of the entire reagent, thus reflecting avidity interactions (Ref. 27). Such an integrated tetramer-based affinity effect is defined as avidity. To ensure the specificity and efficacy of TCR candidates, it is crucial to conduct binding and functional analyses of TCR candidates with pMHC multimers, regardless of their source (Ref. 28).

Functional avidity is an important measure of T-cell activity towards peptide epitopes at different concentrations. The EC50 concentration, representing the peptide dose required to activate half of the T-cell population, is commonly used to describe functional avidity. The concept of functional avidity emerged because the current indicators were insufficient in portraying the extent of TCR activation (Ref. 29). To assess the functional avidity of transduced or native TCRs, different indicators can be used that encompass various aspects of cell function, such as proliferation, cytokine release, cytotoxicity and downstream protein phosphorylation of TCR signalling (Refs 30, 31). To make it more specific, the phosphorylation of linkers, such as extracellular signal-regulated kinase, p38 mitogen-activated protein kinase and nuclear factor of activated T cells 2, can also serve as indicators of functional avidity. Calcium influx, cytokine release (e.g. interferon-*γ* and tumour necrosis factor-*β*) and degree of cell degranulation following stimulation with titrated antigen peptides can also be used (Ref. 32). The most common cytotoxicity ratio obtained by co-culturing TCR-T with tumour cells can also be used. Activation markers such as CD69 and CD137 are upregulated early after CD8 + T-cell stimulation and can also serve as enrichment markers for high-affinity T-cell clones from diverse amplified T-cell subsets from the original library (Ref. 33).

Overall, while pMHC tetramer avidity is a useful tool for identifying TCRs with high-affinity binding to specific antigens, functional avidity assessment provides a more comprehensive evaluation of T-cell reactivity and is a more practical and predictive strategy for screening TCR candidates for adoptive T-cell immunotherapy.

### Theoretical basis and technical strategies for affinity modification

The principle of modifying the affinity of TCRs is based on sound scientific knowledge. The TCR is composed of two chains, *α* and *β*, which combine to form a heterodimeric receptor on the T-cell surface. Each chain consists of a constant domain that anchors the protein in the cell membrane and a variable domain that confers antigen recognition. TCRs interact with pMHC molecules through six complementarity-determining regions (CDRs), as revealed by the binding mode of TCR to pMHC complex (Ref. 26). CDR1 and CDR2 have been identified as binding sites with low affinity to the MHC helix during antigen presentation, although not exclusively (Ref. 34). In contrast, the CDR3 loop, which is responsible for binding peptide antigens presented in the MHC groove, exhibits the greatest genetic variability and diversity as a result of the VDJ recombination process. CDR3*α* and CDR3*β* play a significant role in the antigen specificity (Ref. 35). By employing techniques that enhance the affinity of TCRs, we can increase the affinity value without altering the binding site or antigen specificity recognition on the pMHC surface. Crystallographic experiments demonstrate that the modified TCRs retain good structural stability without any deviation and exhibit excellent safety in various predicted cross-reaction experiments.

At present, the main strategy for enhancing TCR affinity is centred on amplifying the inherent weak affinities by modifying specificity towards the TCR–pMHC interaction. These techniques can be broadly classified into two distinct categories: (1) directed evolution through the use of mammalian, yeast or phage display libraries, and (2) structure-guided computational affinity maturation. These two approaches are complementary and frequently utilized in tandem. Directed evolution involves the use of large TCR libraries, which are fully or semi-randomly displayed on mammalian cells, yeast or bacteriophages, for affinity selection. Soluble pMHC molecules are typically used as targets. Each of these approaches can achieve relatively efficient affinity maturation, with several successful studies having been reported field (Refs 36, 37, 38). However, each of these methods has its own advantages and limitations. When choosing which method to use, factors such as library size, misfolding events, TCR stability and differences between prokaryotic and eukaryotic expression systems must be carefully considered (Ref. 39). By introducing random mutations in the binding region and performing multiple rounds of screening, the hit rates for these methods are typically low. Several rounds of selection are therefore required to achieve sequence convergence and select mutations for further analysis. Careful consideration must be given to which regions to include in the library and mutate to achieve the desired outcome. Libraries designed for mutation and screening in the CDR region primarily focus on the CDR3 region. By introducing mutations in the CDR3 region, high-affinity TCRs can be screened with maintained specificity. Our experience shows that CDR3 fragments show a good safety profile when multiple amino acids act on the binding capacity in experimental validation by alanine scanning experiments. This high-affinity TCR maintains the specificity of the peptide (Refs 3, 37, 40). In our previous understanding of TCR binding modes, compared to CDR3, the CDR1,2 loops are almost completely located on the MHC helix and contribute about two-thirds of the binding free energy, rarely showing direct contact with the peptide (Ref. 41). Therefore, as it is believed that the CDR3 region contributes most to specificity, we seem to be able to increase TCR binding to MHC by changing the CDR1 and CDR2 regions to increase TCR signal activation for a specific peptide. This could then alter the TCR-T response threshold to tumour tissue. The localization of the TCR is such that almost all CDR loops can contribute to the antigenic specificity of T cells (Ref. 42).

Molecular dynamics simulations of TCR–pMHC complexes have been employed using computational methods to explore mutations that could improve binding strength. This approach has been further bolstered by the integration of computational models (Refs 26, 43, 44), which have aided in predicting and assessing binding modes to generate optimized TCR products (Ref. 45). By using deep scanning and analysis based on computational simulations or physically realistic measured structures, researchers can identify key residues and hot spots that can be mutated in combinatorial screens (Ref. 46). In fact, scientists have successfully applied an improved algorithm to redesign a specific TCR and generate mutants based on the structural design that increased affinity up to 400-fold (Ref. 47). This underscores the efficacy of structure-based design in directly targeting the protein interface region of interest and achieving affinity enhancement with only a few amino acid substitutions, which is more efficient than in vitro selection methods (Ref. 48). As more and more pairs of structural information become available and as the accuracy of 3D structure reduction for unknown sequences improves. A combined strategy of these two methods will become the standard for TCR engineering. Structural information of tertiary complexes that provide more molecular detail at the TCR–pMHC interface can be used to select residues or specificities to improve affinity, thereby reducing library size and improving screening efficiency (Ref. 36). Structure-guided methods can be used to add residues to loops and randomize them using display methods, expand specific loops to establish new contacts and potentially increase affinity and specificity (Ref. 49). Whether based on in vitro selection or computationally designed structures, we have been able to increase affinity up to 1 000 000-fold, giving TCR engineering more possibilities.

## Bond energy and functional TCR

### The necessity of introducing other evaluation indicators

In the context of clinical therapy, the predictive role of affinity (be it avidity or functional affinity) for therapeutic efficacy is still limited. For the same pMHC, different TCRs with similar binding affinity can exhibit binding states, which is attributed to the fact that the binding of CDR3 at the TCR–pMHC binding interface exhibits different dynamics (Ref. 50). Typically, non-force conditions are used to measure TCR–pMHC interactions, such as three-dimensional measurements by SPR (Ref. 18). In fact, due to the dynamic nature of T-cell antigen recognition, the TCR–pMHC bond formed at the T cell–APC interface will be subjected to mechanical forces, which inevitably combine with the biochemical aspects of TCR–pMHC interaction to activate T-cell function (Ref. 51). Recent advancements in biophysical techniques have revealed that force enhances TCR–pMHC capture on CD8+ and CD4+ T cells (Ref. 52).

In general, the lifetime of a bond is expected to decrease with increasing force, which is known as a ‘slip bond’. However, it has been observed that bond lifetimes can be enhanced with increasing force, reaching a maximum before exhibiting a similar decrease pattern as slip bonds. This phenomenon is referred to as ‘catch bonds’, which was initially demonstrated in 2003 by Marshall using the adhesive molecule L-selectin (Ref. 53). The force-dependence of the two-dimensional dissociation rate of TCR–pMHC bonds has become a crucial factor in TCR recognition of homologous antigens and TCR triggering (Refs 54, 55, 56).

### Experimental and parameter studies focusing on bond strength

Nowadays, many experiments are available to measure the effect of force on bond lifetimes, such as biofilm force probes, optical tweezers or magnetic tweezers. They have been described in other reviews (Ref. 57). Regardless of the type of assay used, applying force on TCR bonds immediately results in a shortened bond lifetime, forming slip bonds. Alternatively, when the force level increases and the bond lifetime increases before reaching a critical point (peak bond lifetime), catch bonds occur. This is also an important criterion for distinguishing agonist and antagonist antigens. In the case of slip bonds, the parameters that affect this binding form are relatively simple, including the maximum bond lifetime peak and the downward trend of the bond lifetime (Ref. 58). TCRs that exhibit slip bonds also do not exactly exhibit antagonist effects, and such contacts also induce some degree of signal transmission when there are no force interactions at the beginning of the binding.

The TCR produces the effect of a catch bond upon contact with the agonist. Both the force and the bond lifetime required to produce a maximum bond lifetime differ significantly between catch and slip bonds. Thus, both have implications for T-cell activation (Ref. 59). In summary, three important indicators determine T-cell activation type via catch bonds, namely the type of bond, the bond duration at peak and the location of the peak bond lifetime. These parameters offer a superior depiction of the type and intensity of the bonding forces between proteins and serve as a reliable indicator of T-cell reactivity.

The optimal value of each parameter for T-cell activation is currently unknown, and even the optimal degree of T-cell activation has not been determined. However, based on current knowledge, it is understood that a force of approximately ten pN is optimal for signal transduction of T cells upon binding (Refs 60, 61). This conclusion may be limited by the analysis of a relatively small number of TCRs and pMHCs. With the measurement of a larger set of antigens and MHC alleles, differences in force-induced binding lifetimes between T cells, as well as differences in T-cell functional phenotypes, may be revealed.

### Technical solution of bond energy modification

In the realm of TCR recognition, binding is a dynamic process that involves an increase in the total number and stability of atomic interactions or contacts, such as hydrogen bonds and salt bridges, in the stressed state. This enhanced binding is facilitated by various conformational changes at the contact surface, including the exposure of different residues and the proximity of amino acid positions due to rotation, forming new interaction forces that serve as the basis of the catch bond. However, designing directed catch bonds according to this principle poses a challenge, as each TCR–pMHC binding event appears to be unique. Despite the potential benefits, there are several limitations to using computers for predicting and simulating changes in bond energy during binding. One major limitation is the rate of force application to TCR–pMHC complexes, which is many orders of magnitude faster than in-force application experiments. Additionally, the longest simulations currently available are only about 100 ns, which is much shorter than the bond lifetimes observed in the actual process (0.5–5 s). Moreover, no clear structural support has been identified to explain the change in the bond lifetime that occurs upon force application (Refs 62, 63). As a result of these limitations, fixed-point mutagenesis remains the main method for catch bond engineering, with the use of molecular simulations on the computer being considerably constrained.

Catch bond engineering is a biophysical strategy aimed at adjusting the high-sensitivity TCRs used in T-cell therapy while reducing the possibility of adverse cross-reactions. In Garcia's latest engineering method (Ref. 32), it is observed that their method does not significantly differ from the traditional approach based on amino acid mutations. However, their screening work for mutation hotspots is worth noting, as it increases the success rate of modifications by targeting positions that may form new catch bonds. Based on the interaction structure of the TCR and pMHC molecules formed by crystal diffraction, amino acid molecules within a distance of fewer than 4 nanometers (which is almost the maximum distance for two amino acids to interact) are selected for mutation, with a focus on polar and charged amino acids that are more likely to form hydrogen bonds and other interactions with other molecules. At the same time, amino acids with closer interaction distances are excluded, as they may contribute greatly to the specificity of TCR molecules (Ref. 32). Substituting key amino acids in the CDR region can greatly improve the efficiency of modifications (Refs 64, 65, 66). The authors hypothesized that catch bond formation would be most effective if focused on the CDR1 or CDR2 regions, as they believed that the CDR3 region contributes more to specificity. However, catch bond formation is unpredictable, and in some TCRs, the newly formed catch bond may fall within the CDR3 region, while in others, it may be in the CDR1 or CDR2 regions (Ref. 32). Moreover, the effects of catch bond modification at different locations exhibit a non-linear, superimposed effect, which may lead to a plateau in response. This plateau is likely due to the activation of individual T cells being limited (Ref. 32).

The question arises as to whether it is feasible to focus solely on catch bond modifications without considering affinity. Recent studies have shown that catch bonds can recognize low concentrations of antigens: It was observed that the dose did not change the degree of activation of antagonist and partial agonist antigens on T cells (Refs 67, 68). This suggests that modifications targeting catch bonds alone may be sufficient, as opposed to modifications targeting affinity. In summary, catch bond mutation modifications can enhance T-cell responsiveness without increasing affinity, thereby mitigating potential cross-reactivity issues. Traditional affinity-based modifications can easily produce a large number of TCRs with high affinity, leading to significant variations in responsiveness and off-target effects. Nonetheless, there are still conflicting situations and unexplained phenomena regarding TCR responsiveness. Moreover, current findings did not eliminate the role of affinity in TCR responsiveness, as the yeast surface display experiment used to predict peptide reactivity and evaluate safety was still based on the affinity (Ref. 32). Therefore, the author's article provides a better parameter for TCR responsiveness but does not change the process of improving responsiveness by first increasing affinity. Future research should identify the structural basis for easily forming catch bonds to enable more efficient TCR modifications.

### Focus on the new content of the bond modification

Glycosylation is a catalytic process that is mediated by enzymes, leading to the formation of glycosidic bonds between carbohydrates and other compounds. Along with protein hydrolysis and disulphide bond modifications, glycosylation represents a significant post-translational modification that results in a diverse and intricate polysaccharide repertoire (Ref. 69). In addition to influencing T-cell activity and signalling, glycosylation plays a vital role in regulating key physiological and pathological aspects of T-cell biology, including T-cell differentiation, proliferation, thymocyte selection and development. These modifications are critical in determining the interactions between tumour cells and human T cells (Ref. 69). When it comes to immunity, polysaccharides are essential in almost all signal and cell interactions, especially in ligand–receptor interactions (Ref. 70). Glycosylation is involved in various processes of T cells and directly influences the strength of TCR binding. In designing protein-based TCRs, we focus on their role in TCR binding.

A recent study examined a pair of TCR–pMHC molecules found in melanoma and discovered that the addition of a glycosyl group to the N-terminal end not only altered the conformation of the protein but also prolonged the bond lifetime. During the binding and separation process, the increased number of hydrogen bonds and Lennard–Jones contacts further emphasized the effectiveness of glycosylation as a post-translational modification (Ref. 71). These findings demonstrate the importance of N-glycosylation in TCR–pMHC binding and highlight the need for its consideration in future experimental and computational studies. Glycosylation of transmembrane surface proteins plays a pivotal role in various biological processes. CD43, CD45 and CD25 are among the most abundant glycoproteins found on the surface of T cells and are expressed at all stages of T-cell development. They are decorated with O- and N-glycans and their glycosylation is involved in various processes such as activation, differentiation and apoptosis, which greatly affect the T-cell receptor signalling (Ref. 72). Further studies are needed to explore other transmembrane proteins that may also be highly correlated with glycosylation.

Currently, glycosylation engineering faces several significant limitations, and precise manipulation of the glycome for specific proteins in living cells remains unachievable using current technology. Instead, current approaches are only capable of adjusting the overall glycosylation level of proteins on the cell surface. A wealth of empirical evidence supporting the utility of glycoengineering can be found in the extensive studies on anti-cancer antibodies, which have demonstrated the efficacy of glycoengineering in enhancing the therapeutic potential of the protein biopharmaceuticals (Ref. 73). Various strategies for designing glycans to confer new properties and functions to therapeutic cells include genetic engineering approaches, metabolic glycoengineering and chemoenzymatic glycoengineering. Interested readers can find a comprehensive review on the subject in the following reference (Ref. 74).

## Armed TCR-T

### The impact of co-receptor on response

**Figure 1.**
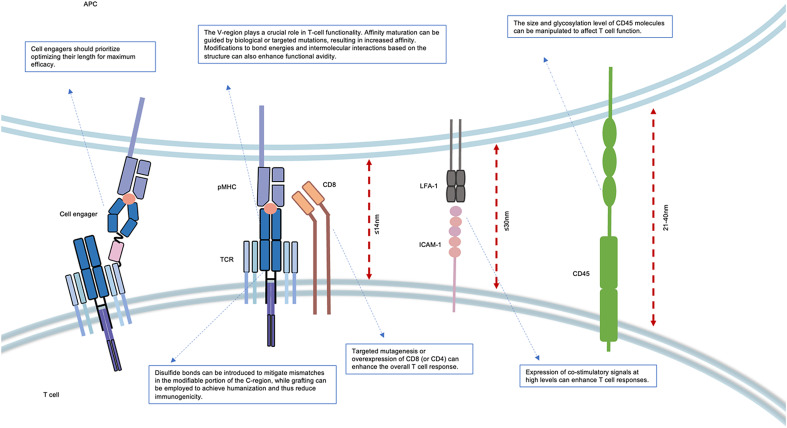
The T-cell structure interacts with antigen-presenting cells (APC) and forms immune synapses. T-cell response is influenced by factors such as V and C regions of TCR, co-stimulatory molecules (CD4, CD8) and CD45 molecules. Hence, all aspects of T-cell function can be modified and engineered to enhance its function. IMMTAC (immune mobilizing monoclonal T-cell receptor against cancer) is a fusion protein comprising a soluble TCR on one end, recognizing the pMHC complex and targeting tumours. An anti-CD3 scFv (single-chain variable fragment) on the other end, which recognizes CD3 molecules to activate T cells. The interaction between pMHC (peptide-major histocompatibility complex) and TCR (T-cell receptor) necessitates the participation of auxiliary molecules, which assemble at the core of the immune synapse. Concurrently, co-stimulatory molecules and CD45 are situated in the outermost layer of the immune synapse, and the extent of T-cell activation is determined by their collective size. Modifications to the TCR can involve both V and C region. Modification of the V-region can enhance affinity and bond strength, while modification of the C-region can reduce immunogenicity and decrease TCR mismatches. Overexpression and modification of the helper molecule CD8 can augment the T-cell response. Elevating the levels of co-stimulatory molecules and increasing the length or altering the glycosylation of CD45 in the outermost layer of the immune synapse can also boost T-cell activation.

MHC ligands have the ability to interact with co-receptors, such as CD4 or CD8, in addition to the TCR (Fig. 1). This makes it challenging to measure TCR affinity on the T-cell surface, as these additional binding interactions can complicate the binding strength between pMHC and TCR and the extent of lymphocyte-specific protein tyrosine kinase (LCK) recruitment to the TCR complex for downstream signalling cascades, which can vary (Refs 75, 76).

CD4 co-receptors have a weak KD (150–200 *μ*m) and exhibit rapid kinetics when bound to class II MHCs. Its contribution to TCR–pMHC interactions is negligible (Ref. 76). It is also believed to have minimal impact on MHC tetramer binding (Ref. 21). Although this does not suggest that CD4 lacks importance in functional responses, as it plays a vital role in the recruitment of LCK to optimize T-cell signalling (Ref. 77).

CD8 has been shown to augment the binding and dissociation rates of MHC tetramer binding, leading to an increase in the affinity and stability of T cells' interaction with their cognate multimeric ligands (Ref. 78). While the affinities of both CD8 and CD4 for their coreceptors are relatively weak, CD8 exhibits a higher affinity than CD4. Removal of CD8 contribution leads to a decrease in both affinity and functional response fields (Ref. 79). Additionally, CD8 deletion has a greater impact on the binding of T cells with lower affinity, indicating that CD8 enhances the probability of low-affinity TCR–pMHC interactions and signal transduction. These TCRs are known as CD8-dependent, and their affinity threshold range is between 60 and 120 *μ*M (Ref. 80). Several CD8-independent TCRs with higher affinity have been identified in established TCR products, and their affinity ranges from 4 *μ*m to 26 pM (Ref. 4). However, the specific binding threshold that confers CD8 independence to a TCR remains undefined (Ref. 81).

Based on the aforementioned facts, the elimination of CD8 co-receptor binding to the MHC molecule binding region can block T-cell-mediated lysis of specific target cells without disrupting the TCR's specific interaction with the MHC (Ref. 82). Conversely, artificial modifications to MHC molecules that enhance CD8 binding have the potential to promote T-cell proliferation and cytokine release (Ref. 68). Nevertheless, clinical trials involving the overexpression of these molecules have shown a response rate increase of only 40–60%, and the effect appears to plateau, suggesting the limited potential for coreceptor and accessory molecule modification (Refs 61, 83). Modification of co-receptors is particularly important for low-affinity TCRs, as an increasing number of low-affinity TCR-T products have been found to possess potent antitumour effects, underscoring the significance of co-receptor modification as a critical consideration in therapeutic development.

### Co-stimulatory inhibitory molecules and TCR activation signalling pathways

The initial step in activating downstream signalling pathways is the binding of TCR to MHC, where the formation of immunological synapses between T cells and target cells serves as the foundation for immune responses (Ref. 84). Besides the central site of TCR and MHC binding, there exist numerous co-stimulatory signalling receptor interactions at their periphery that regulate signal strength. Early theories of T-cell activation proposed that complete activation of LCK was a prerequisite for TCR signalling (Ref. 85). Furthermore, it has been discovered that full T-cell activation necessitates the matching of tumour ligands with independently triggered costimulatory receptors, as well as overcoming inhibitory receptor signalling expressed in the TME (Ref. 86). CD28 is the most classical molecule that recruits CD8 or CD4 co-receptors (Ref. 85). The cytoplasmic structural domains of CD4 and CD8 are capable of recruiting tyrosine kinase LCK and mediating immunoreceptor tyrosine-based activation motif phosphorylation in the cytoplasmic structure of the CD3 subunit (Ref. 72). Subsequent recruitment of Zeta-chain-associated protein kinase 70 to the TCR further promotes the LCK activation (Ref. 86).

**Figure 2.**
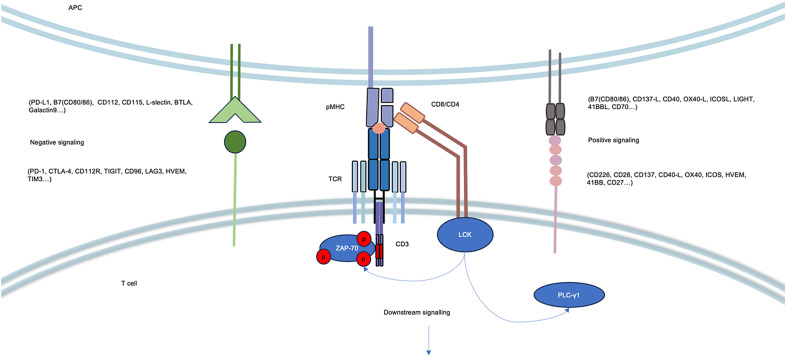
Various co-receptors exist between TCR and tumour cells/APCs that transmit positive and negative signals. The cytoplasmic domains of CD4 and CD8 recruit the tyrosine kinase LCK and mediate the phosphorylation of the immunoreceptor tyrosine-based activation motif (ITAM) in the cytoplasmic structure of the CD3 subunit, and CD28 is the most classical molecule of the co-receptor recruited by either CD8 or CD4, and the full activation of LCK is a prerequisite for TCR signalling. Recruitment of ZAP-70 to the TCR further promotes LCK activation, and the TCR signalling pathway is then maintained by phospholipase C-gamma 1 (PLC-*γ*1). In addition to the first signals generated by TCR binding to pMHC, T-cell activation requires many other co-receptors to provide positive signals to maintain the activated state to promote killing, as well to regulate the degree of T-cell activation, or tumour cells to implement immune escape. A series of negative signals exist between the T cell and the tumour cell.

There are a number of complex co-receptors that regulate immune actions in the interaction of T cells with APC/tumour cells, including positively regulated receptors and ligands that promote T-cell activation, as well as negatively regulated factors that modulate T-cell function and lead to immune escape from tumours (Fig. 2). A deeper understanding and knowledge of these modes of regulation will help us design better ARMED TCR-T cell therapy products (Ref. 87) (Table 1). CD28, 41-BB, inducible T-cell co-stimulator and CD134 are classic co-stimulatory receptor combinations. The incorporation of cellular co-stimulatory signalling domains into next-generation CAR-T cell design has been demonstrated to be critical for their functionality (Ref. 73). Similarly, in clinical TCR-T cell therapy, enhanced co-stimulatory signalling can promote cell proliferation, cytotoxicity and cytokine production (Ref. 88). However, in the process of modifying and optimizing these cells, the specific co-stimulatory signalling scheme needs to be refined in many aspects. Firstly, the incorporation of co-stimulatory domains affects the overall cell function, including cell differentiation state and metabolism level. The selection of different costimulatory molecules can allow T cells to exhibit different phenotypes, such as a central memory phenotype (Ref. 89). Secondly, there are challenges in the transfer of co-stimulatory molecules into cells, although many switch receptors exhibit good effects in vitro and in vivo. When we introduce additional ligands on cells, these ligands with exogenous TCRs can be inserted by a large vector or two independent vectors, which limits the transmission efficiency (Ref. 90). Thirdly, an excessive increase in the intensity of the co-stimulatory signal may lead to unexpected effects, as evidenced by the clinical effects of third-generation CAR-T cells, where incorrect combinations of co-stimulatory domains can trigger cytokine storms and T-cell tolerance, greatly affecting the safety and efficacy of cell therapy (i.e. rapid T-cell exhaustion may occur) (Ref. 77). These findings suggest the necessity of designing TCR-T therapies based on the co-stimulatory signalling basis.

**Table 1.** List of published ARMED TCR-T or CAR-T cell clinical trials

Target antigen	Retrofitting method	Epitope	Cancer type	Number of patients	Clinical trial	Phase	Objective response rate (ORR) (%)	Toxicities related to T-cell therapy(%)
MART-1	Affinity maturation	AAGIGILTV	Melanoma	20	NCT00509288	II	6 (30%)	Skin rash: 14 (70%)
MART-1	Optimization sequence	EAAGIGILTV	Melanoma	13	NCT00910650	II	0	CRS: 2 (15%)
MART-1	Optimization sequence without affinity maturation	EAAGIGILTV	Melanoma	12	NCT02654821	I/IIa	2 (16.7%)	CRS/sepsis: 1 (8%)
gp100	Affinity maturation	KTWGQYWQV	Melanoma	16	NCT00509496	II	3 (16%)	Skin rash: 15 (94%)
CEA	Affinity maturation	IMIGVLVGV	Colorectal cancer	3	NCT00923806	I	1 (33%)	Severe transient colitis: 3 (100%)
MAGE-A3	Affinity maturation	KVAELVHFL	Melanoma	9	NCT01273181	I/II	5 (56%)	Severe neurologic toxicity: 3 (33%)
MAGE-A3	Affinity maturation	EVDPIGHLY	Melanoma	2	NCT01350401	I	0	Severe cardiac toxicity: 2 (100%)
MAGE-A3	Affinity maturation		Metastatic solid tumours	17	NCT02111850	I	4 (23.5%)	Liver toxicity: 2 (12%)
MAGE-A4	Affinity maturation	NYKRCFPVI	Oesophageal cancer	10	UMIN000002395	I	0	
MAGE-A4	Affinity maturation	GVYDGREHTV	Advanced solid tumours	38	NCT03132922	I	9 (23.7%)	CRS: 9 (50%)
MAGE-A10	Affinity maturation	GLYDGMEHL	NSCLC	11	NCT02592577	I	1 (9%)	CRS: 3 (27%) Neurotoxicity: 1 (9%)
NY-ESO-1	Affinity maturation	SLLMWITQC	Melanoma	17	NCT00670748	I	5 (45%) 4 (67%)	None
NY-ESO-1	Optimization sequence	SLLMWITQC	Melanoma	38	NCT00670748	II	11 (55%) 11 (61%)	None
NY-ESO-1	Affinity maturation	SLLMWITQC	Synovial sarcoma	12	NCT01343043	I/II	6 (50%)	CRS: 5 (42%)
NY-ESO-1	Affinity maturation	SLLMWITQC	Synovial sarcoma	30	NCT01343043	I/II	9 (30%)	n.s.
HPV16-E6	Affinity maturation	TIHDIILECV	HPV16-positive epithelial cancer	12	NCT02280811	I/II	2 (17%)	None
HPV16-E7	Natural sequence	YMLDLQPET	HPV16-positive epithelial cancer	12	NCT02858310	I	6 (50%)	None
HBV	Affinity maturation	N.A.	HBV-HCC	8	NCT03899415	I	1	Liver toxicity: 1 (12%)
MCPyV	Affinity maturation	KLLEIAPNC	Merkel cell carcinoma	5	NCT03747484	I	1 (25%)	None
TP53	Affinity maturation	HMTEVVRHC	Metastatic breast cancer	1	NCT03412877	I	1 (100%)	CRS
KRAS G12D	Affinity maturation	GADGVGKSA	Metastatic pancreatic cancer	1	IND 27501	I	1 (100%)	None

CEA, carcinoembryonic antigen; gp100, glycoprotein 100; HPV, human papillomavirus; MAGE-A, melanoma-associated antigen; MART-1, melanoma antigen recognized by T cells 1; NY-ESO-1, New York oesophageal squamous cell carcinoma-1; NSCLC, non-small cell lung cancer; CRS, cytokine release syndrome; n.s., not specified; KO-KI, knockout–knock-in; N.A., not available.

Most of the TCR-T products that entered clinical testing in the early days underwent a process of sequence optimization and affinity maturation, with only a very few using natural TCR sequences directly. Due to the inconsistency of standards and some technical details have not been missed. Some articles only briefly describe their TCR for sequence optimization. Thus, in this sequence, optimization mainly represents a modification of the natural TCR sequence by the authors, with most of the modifications focusing on affinity maturation. Those that performed sequence optimization without increasing affinity have been specifically labelled.

Interfering with the downstream signalling pathways of T cells is a promising avenue for therapeutic modification. The initial TCR signalling pathway is maintained by phospholipase C-gamma 1 and vitamin D receptors, both of which are crucial for the classical TCR signalling pathway (Ref. 91). Subsequently, the pathway increases intracellular Ca2+ levels and activates T-cell-associated binding proteins such as calmodulin and activated T-cell nuclear factor protein 2, as well as nuclear factor proteins, which induce T-cell activation and the release of large amounts of cytokines and chemokines (Ref. 78). Modulation of the signalling pathways underlying T cells can directly regulate the function of the T cells, making it highly applicable to any tumour epitope, and can be easily replicated. Such modifications are more direct and effective than affinity and binding modifications. However, further experiments are required to determine the optimal T-cell activation strength and signalling state to effectively execute response-adapted cytotoxic functions in the TME.

### Strategies for overcoming the transformation of tumour microenvironment

The TME has emerged as a pivotal determinant in both the advancement of cancer and the shortcomings of therapeutic interventions. Nestled within a heterogeneous milieu constituted by infiltrating and resident host cells, secretory factors and the extracellular matrix, tumour cells find their abode (Ref. 92). Among the infiltrating immune cell contingent, T cells (including tumour-infiltrating lymphocytes and regulatory T cells), macrophages (M1 and M2 phenotypes) and myeloid-derived suppressor cells (MDSCs) play vital roles (Refs 92, 93).

Concurrently, the TME is pervaded by secreted immune-suppressive cytokines such as IL-10 and TGF*β*, as well as tumour-promoting chemokines. Among the components of the extracellular matrix, stromal cells, including cancer-associated fibroblasts (CAFs) and tumour-associated macrophages (TAMs), exert their influence (Refs 93, 94). In stark contrast to haematologic malignancies where T cells can readily infiltrate owing to the robust exposure to tumour antigens, the immunosuppressive factors inherent in the TME pose formidable challenges. Transferred T cells frequently exhibit functional deficits or swift exhaustion upon successful penetration (Ref. 95). Consequently, in the context of solid tumours, the myriad inhibitory factors within the TME emerge as inescapable obstacles that undermine cell-based therapies; nevertheless, they present themselves as prime candidates for therapeutic targeting. One noteworthy target in this landscape is fibroblast activation protein (FAP), an enzyme endowed with dual proteolytic activities. FAP is found to be overexpressed in CAFs across various tumour types, while its expression is sparse in healthy adult tissues. By harnessing FAP as a target, engineered T-cell therapies such as TCR-T and CAR-T could be tailored to specifically address the tumour stroma, thereby enhancing T-cell infiltration. Encouraging therapeutic outcomes have already been observed in diverse solid tumours through the deployment of FAP-targeting CAR-T cells (Ref. 96).

In the intricate meshwork of TME, most cell types interact with T cells, interacting through immune checkpoints such as the PD-L1 ligand, leading to the suppression of anti-tumour activity and consequent T-cell exhaustion. Cells implicated in this interplay encompass cancer cells as well as infiltrating monocytes, including dendritic cells (DCs) and macrophages (Ref. 97). Furthermore, activated T cells expressing CTLA-4 exhibit enhanced affinity for binding to CD80/86 on APCs, effectively outcompeting the binding of co-stimulatory molecule CD28 and thus impeding anti-tumour immune function (Ref. 98). Such inhibitory signals are more extensively expounded upon in Figure 2. Nonetheless, monotherapy with immune checkpoint inhibitors (ICBs) often yields unsatisfactory clinical outcomes (Refs 99, 100).

Addressing the impediments posed by the TME to the efficacy of TCR-T and CAR-T therapies holds significant potential. A compelling approach involves the utilization of CRISPR-CAS9 technology to introduce a chimeric switch receptor (CSR) comprised of the extracellular domain of PD-1 and the intracellular domain of CD28 (Refs 101, 102). Rather than propagating inhibitory signals, this interaction transmits co-stimulatory signals through CD28. Encouragingly, this strategy has demonstrated exceptional therapeutic efficacy in clinical investigations, all without evidence of significant neurotoxicity or cytokine release syndrome. Additionally, CAR-T cells targeting CTLA-4 have also exhibited noteworthy therapeutic impact, with diverse adaptations of this design remaining under exploration (Ref. 103). A notable example involves the fusion of the extracellular domain of Fas with the intracellular domain of 4-1BB via CSR. This reconfiguration effectively transforms the Fas ligand-mediated death signal into a survival-promoting signal (Ref. 104).

Expanding the strategies to enhance T-cell infiltration into the TME introduces a novel approach – augmenting the expression of chemokine receptors on tumour-specific cytotoxic T lymphocytes and targeting tumour-secreted chemokines (Ref. 89). Within this context, the versatile array of chemokine ligands for the CXCR2 receptor is prominently expressed in a multitude of tumours (Ref. 105). These chemokines, including CCL2, CCL7 and CCL8, are also detected within the TME, particularly in cancer cells, CAFs, TAMs, MDSCs and stromal stem cells. These chemokines play a pivotal role in supporting tumour growth and dissemination (Ref. 90). In light of this, the targeting of CCR2 and CXCR2 receptors has gained substantial traction as a therapeutic avenue within cancer immunotherapy. In the realm of TCR-T therapies, introducing CXCR2 to pmel-1 TCR transgenic T cells or MAGE-A3-specific TCR-engineered T cells has demonstrated tangible benefits (Refs 106, 107). CXCR2-TCR-T cells exhibit enhanced homing to tumours, heightened tumour infiltration and preferential accumulation at tumour sites in comparison to control TCR-T cells. This phenomenon is also observed in the context of CAR-T therapies (Ref. 108). Collectively, these findings underscore the potential of introducing chemokine receptor genes into tumour-specific T cells to amplify their tumour homing and localization capabilities, ultimately improving antitumour immune responses (Ref. 109).

Moreover, T cells can be engineered to overexpress chemokines and cytokines, further elevating their antitumour efficacy. For instance, the transduction of CAR-T cells to express IL-7 and the chemokine CCL-19 not only enhances T cell survival, infiltration and accumulation within tumours but also culminates in the complete regression of established solid tumours and extended survival in murine models (Ref. 110). By integrating chemokines such as CCL21 and IL-7 into CAR-T cells, a substantial augmentation in the survival and infiltration of CAR-T cells and DCs within tumours is observed, leading to complete tumour remission (Ref. 111). This multifaceted approach highlights the potential to harness the dynamics of chemokine receptors and their ligands to bolster T-cell infiltration, survival and antitumour responses within the complex milieu of the TME.

## TCR-T safety assessment

### Humanization, mismatch challenges and off-target effects

One of the defects of TCR engineering is the low expression caused by mismatching between transgenic TCR and natural TCR. The emergence of novel cross-reactive features resulting from a potential discrepancy between the endogenous *α*-stranded *β*-strand and the exogenous foreign-assisted TCR receptor introduced in TCR gene therapy constitutes a possible cause of off-target events (Ref. 1), especially for human-derived TCRs, which have a high probability of mismatch. To address this issue, strategies mainly focus on changes in the constant region, including replacing human constant regions with mouse constant regions, minimal monetization, codon optimization, adding disulphide bonds, introducing hydrophobic mutations, removing N-glycosylation sites, TCR domain swapping, etc. (Refs 112, 113). Other strategies include expressing transgenic TCRs as TCR-CD3*ζ* fusion proteins, using single-chain TCR formats, or silencing or knocking out endogenous TCR chains (Refs 114, 115, 116). None of these methods can avoid the production of mismatched TCRs. Introducing non-human-derived TCRs can better solve this problem because they cannot bind with CD3, making mismatches ineffective (Ref. 112). Although the anti-tumour function of TCRs is enhanced, the presence of xenogenic materials may lead to immunogenicity and hinder the effectiveness of cells. This problem can be solved by replacing key residues in the constant region of TCR with mouse-derived residues (Ref. 117). Therefore, TCRs or antibody products inevitably require humanization modification.

The success of humanized antibodies, which mitigate the immunogenicity of mouse-derived antibodies, provides a basis for current knowledge. These antibodies have exhibited remarkable success in clinical trials (Refs 118, 119). The strategy behind humanizing mouse-derived antibodies involves utilizing human antibodies as a backbone and transplanting the minimal functional region from the mouse-derived look that confers antigenic specificity, that is, the CDR, into the corresponding CDR region of the human antibody (Ref. 120). Despite the antigen-binding effect being preserved to some extent, the affinity values usually undergo changes (Ref. 121). This may be attributed to the alteration in CDR conformation after transplantation into the human frame (Ref. 122). Such phenomena are primarily observed in the structures of antibody-based receptors, such as chimeric antigen receptors found in T cells from mice (Ref. 112). Drawing on the experience of constructing humanized antibodies, successful cases have been achieved by transplanting CDRs from mouse TCRs into human variable compartments to reduce immunogenicity. To enhance the stability of the CDR framework post-transplantation, computer modelling is utilized to introduce point mutations that optimize key interactions (Ref. 123). It has been discovered that modifying the TCR framework on variable regions that do not contribute to antigen recognition can safely enhance the affinity of TCR-transgenic T cells (Ref. 115).

### The use of external control systems to ensure safety

In order to control off-target effects and the occurrence of autoimmune diseases in clinical settings, we may introduce an exogenous control system. Although many unknown factors still exist, we can still develop safe engineering of therapeutic T cells, as well as drugs with controllable switches to rapidly eliminate T cells in vivo (see review (Ref. 124)). Three main strategies have been studied clinically: (1) the herpes simplex virus thymidine kinase (HSV-TK) suicide gene, derived from the herpes simplex virus type I (HSV-TK), is one of the most common suicide genes. HSV-TK has shown good safety in cell-based immunotherapy but requires the introduction of phosphorylated nucleoside analogues. (2) Inducible caspase-9 (iC9) can also be used as a safety switch for induced T cells. iC9 is a modified human FK binding protein that can be activated by a small molecule AP1903, which depends on the mitochondrial apoptotic pathway. The immunogenicity of iC9 suicide genes is lower, leading to a decrease in immune reactions against transgenic cells. The inducible caspase 9 (iCasp9 or iC9) suicide gene is a safety switch mechanism for TCR-T cells. Another strategy to control the toxicity of TCR-T cells is to implant ‘off-switches’ or ‘suicide genes’ into the TCR structure. Another strategy that dynamically and rapidly controls CAR-T cell function is based on protease-assisted small molecule shuttles for CARs, also known as switching CARs off with inducible fast-off. These small molecules can be used to modulate the expression of CARs on the surface of T cells. In this system, the protease cleavage site and the degron domain are embedded together in the CAR construct, and the degron domain promotes the degradation of CAR protein. In the ‘on’ state, the cleavage site is cut, resulting in the removal of the degron from the CAR protein, which is then expressed on the surface of T cells. However, when using small exogenous molecule protease inhibitors, the CAR protein is not cleaved, resulting in the retention of the degron, which is then degraded through proteolytic pathways, leading to the degradation of CARs.(3) Cell Elimination Tags (CET) (Ref. 124). Carcinoembryonic antigen (CEA) Tmod cells, which have been clinically evaluated, can also be used to alleviate TCR-T clinical toxicities. CEA Tmod cells use two receptors: a CAR activated by CEA and an inhibitory receptor based on leukocyte Ig-like receptor 1 triggered by human leukocyte antigen. Because there is a phenomenon of human leukocyte antigen (HLA) heterozygosity loss in tumour cells, CEA Tmod cells are highly selective against tumour cells expressing CEA and effectively kill them (Ref. 125). TCR cells can also refer to CEA Tmod cells and express both receptors to specifically recognize tumour cells while avoiding recognition of normal cells in the body. Target cell depletion is achieved through antibody-dependent cell cytotoxicity (Refs 126, 127, 128). Several clinical trials of CET are ongoing, but formal proof of their clinical impact on toxicity control is still pending.

## A new generation of products based on T-cell receptor

Over the past decade, numerous breakthroughs in cancer immunotherapy have emerged, all of which involve the regulation of T-cell-mediated immunity. Currently, there are three major types of cancer immunotherapies approved for clinical use: (i) ICBs, (ii) genetically engineered T cells expressing CARs and (iii) bispecific antibodies (bsAbs). New types of therapeutic cells and products have been designed for immunotherapy with various styles. Based on identified patterns, these new products can be classified into two types based on their binding patterns: (1) products based on antigen–antibody binding patterns and (2) products based on the TCR and pMHC binding patterns. These products can be further classified into antibody-based and cell-based forms. Representative examples of these products include bispecific T-cell engager (BiTE), cell engager, TCR-like antibody (TCRL ab), synthetic TCR and antigen receptor T-cell, bispecific or multispecific CAR-T and TCR-T.

### Cell engager and TCRL antibody

The recognition patterns of Abs and TCRs differ, yet both possess diversity in receptor binding sites and specificity for antigen recognition fields (Refs 129, 130). Like TCR alpha/beta heterodimers, Fab H/L heterodimers directly contact homologous antigens using two sets of CDRs, also known as variable fragments. TCRs exert sub-micromolar affinity for homologous pMHC when acting on cells (Ref. 131), while Abs possess nanomolar affinity and interact with their specific antigen, with binding energies over 100-fold higher (Ref. 132). The collective concentration of antibody-type drugs is bound by the picomolar affinity (Ref. 133). Such vast differences necessitate affinity optimization and engineering when converting TCRs to soluble TCRs, a process fraught with significant technical difficulties. This phenomenon is well understood, given the contributions of T-cell triggering mentioned earlier, such as dissociation from various accessory molecules and TCR clustering.

In order to address this issue, scientists have pursued two approaches. Firstly, researchers have developed TCRL monoclonal antibodies (mAbs) by combining the high affinity of mAbs with the ability to recognize pMHC complexes (Ref. 134). However, others have recently conducted an in-depth investigation of the specific binding mode of TCR-like antibodies, comparing them to cell engager and simulating their binding in a manner highly like natural TCRs. However, the structural analysis revealed that half of the peptide contacts produced by these antibodies interacted with different residues than those contacted by TCRs and instead made additional crucial contacts with other peptide residues. This structural feature, which differs from the peptide-binding hotspots of natural TCRs, is associated with self-reactive TCRs and may be related to high levels of TCR cross-reactivity (Ref. 135). Thus, it is concluded that the core region of the target molecule is susceptible to distortion upon binding by antibodies to pMHC (Ref. 50).

Another direction of exploration is to preserve the original TCR recognition structure and segments while optimizing and modifying the affinity of TCRs by making them soluble. Cell engagers are a type of fusion protein, akin to bsAbs, that feature a soluble TCR on each end and an anti-CD3 Single-chain variable fragment (scFv) structure that activates T-cells. The TCR end selectively targets the tumour site, while the single-chain antibody fragment confers the corresponding functional properties. Unlike conventional antibodies, which only target extracellular antigens and secreted proteins on the cell surface, the TCR recognition structure is based on the ability of human leukocyte antigens to target peptides presented by HLA molecules as intracellular targets (Ref. 136). This mechanism directs immune cells to eliminate cancer cells (efs 137, 138). The high picomolar affinity of the TCR for pMHC has enabled the packaging of cell engagers into target cells. The recent launch of IMMTAC(Immune Mobilizing Monoclonal T-cell Receptor Against Cancer) by IMMUNOCORE has certainly given a boost to this type of product (Ref. 139). However, the structure of soluble TCRs does not exist under physiological conditions, and the modification of soluble TCRs has taken a long time. Moreover, after obtaining stably expressed soluble TCRs in vitro, we still need to increase their affinity several orders of magnitude to make them effective in targeting. However, for TCRL mAbs, such binding seems to be more in line with the binding pattern under physiological conditions and more stable. Therefore, we can speculate that affinity modification based on the natural TCR receptor seems to be safer.

### The determinative role of length

The formation of the immunological synapse is fundamental for T-cell activation and function, and the mode of TCR–pMHC molecular binding is mainly explained by the kinetic segregation model. The spatial rearrangement of proteins at the cell–cell interface is a crucial principle for understanding the receptor activation (Refs 140, 141). In the natural TCR binding mode, this range of sizes encompasses a spectrum extending from the intermembrane spacing of the native TCR–pMHC complex, which is approximately 14 nm (Refs 41, 142), to sizes surpassing the spacing of roughly 30 nm ascertained by the binding of intracellular adhesion molecule 1 to lymphocyte function-associated antigen 1 (Ref. 143), and the large extracellular domain of CD45, which ranges from 21 to 40 nm (Refs 144, 145). For some time, the size of the TCR–pMHC complex at T cell–APC junctions has been thought to preclude the binding of CD45 due to steric hindrance (Refs 144, 146, 147) Therefore, specific binding distances are required to achieve better efficacy in this binding mechanism (Fig. 1). In experiments where we altered the length of the TCR–pMHC binding at the centre of the immunological synapse, we found that the intermembrane distance established during TCR binding events could affect ligand potency, which is far greater than the effect of epitope selection (Refs 148, 149).

The fundamental basis of T-cell function lies in specific spatial distances. Deviation from appropriate distances can disrupt the formation of immune synapses, leading to a significant reduction in T-cell activation. This discovery provides valuable insights into the optimization of natural TCR-T cell therapies. In the context of CAR-T cell therapy, the axial spacing between membranes can be manipulated to improve efficacy (Ref. 150). Artificially designed CAR-T cells have been extensively studied and validated, revealing that shorter membrane spacing induced by CAR antigens results in stronger CD45 rejection, leading to increased mobilization of CAR molecules to the membrane-proximal epitopes of structural domain 7, located approximately 2 nm from the membrane (Refs 151, 152). These findings suggest that closer proximity between cells, similar to the distance between natural TCR and pMHC complexes or even shorter, can trigger greater T-cell activation. As the length of the CAR increases, the repulsive effect of CD45 on CAR synapses diminishes (Ref. 153). Bispecific CAR-T cells also exhibit weaker responses to molecules located at greater distances than those located in closer proximity, further highlighting the importance of spatial distance in cellular responses (Ref. 154).

The results of the experiment demonstrate that the extracellular domains of CD45 RABC and CD45 RO（one of the four major isoforms of CD45）exhibit different sensitivities to the distribution force generated by ligand–receptor binding at the membrane–membrane interface (Ref. 155). This finding is consistent with previous observations indicating that T-cell signalling is more effective when expressing larger CD45 isoforms and confirms the biophysical differences between the two highly conserved CD45 subtypes with respect to their spatial segregation in response to TCR–pMHC interactions (Refs 155, 156). Based on this, an alternative approach to enhancing T-cell responsiveness can be achieved by manipulating the relative sizes of the TCR–pMHC complex and CD45 molecule, revealing that CAR-T activation depends on the difference in size between the CAR antigen and CD45, and using anti-CD45 antibodies as spacers can not only enhance the activation of CAR-T cells under physiological conditions but also restore the efficacy of long scFV CAR-T cells that were previously impeded by excessive synapse centring (Ref. 157). Thus, the size of CAR, antigen and CD45 can serve as targets for modulating CAR-T activation.

Thus, in CAR-T and artificial T-cell activation structures, such as cell engager, spatial distances are often overlooked, leading to the failure of T-cell response. These findings are consistent with previous studies of bispecific T-cell engagers that share similar structures, which indicate that these TCR ligands are more effective when they bind epitopes that create smaller intermembrane spaces, thereby bridging TCRs on a T cell to melanoma markers on opposing cell. Therefore, our understanding of the important role played by spatial distance in T-cell response highlights the need for incorporating this parameter into the design of various cell engagers, including bispecific or multispecific TCRs, such as T-cell receptor fusion constructs (Refs 158, 159), and other possibilities mentioned in this article (Ref. 158). By taking this parameter into account during the initial design phase, we can predict the reactivity of these structures more accurately.
